# Dapagliflozin is associated with lower risk of cardiovascular events and all‐cause mortality in people with type 2 diabetes (CVD‐REAL Nordic) when compared with dipeptidyl peptidase‐4 inhibitor therapy: A multinational observational study

**DOI:** 10.1111/dom.13077

**Published:** 2017-09-08

**Authors:** Frederik Persson, Thomas Nyström, Marit E. Jørgensen, Bendix Carstensen, Hanne L. Gulseth, Marcus Thuresson, Peter Fenici, David Nathanson, Jan W. Eriksson, Anna Norhammar, Johan Bodegard, Kåre I. Birkeland

**Affiliations:** ^1^ Steno Diabetes Centre Copenhagen Denmark; ^2^ Karolinska Institutet, Södersjukhuset Stockholm Sweden; ^3^ Oslo University Hospital Oslo Norway; ^4^ Statisticon AB Uppsala Sweden; ^5^ AstraZeneca Cambridge UK; ^6^ Uppsala University Uppsala Sweden; ^7^ Karolinska Institutet Stockholm Sweden; ^8^ Capio S:t Görans Hospital Stockholm Sweden; ^9^ AstraZeneca Nordic‐Baltic Oslo Norway; ^10^ University of Oslo Oslo Norway

**Keywords:** cardiovascular disease, dapagliflozin, diabetes complications, DPP‐4 inhibitor, hypoglycaemia, type 2 diabetes

## Abstract

**Aims:**

To compare the sodium‐glucose‐cotransporter‐2 (SGLT‐2) inhibitor dapagliflozin with dipeptidyl peptidase‐4 (DPP‐4) inhibitors with regard to risk associations with major adverse cardiovascular (CV) events (MACE; non‐fatal myocardial infarction, non‐fatal stroke or cardiovascular mortality), hospitalization for heart failure (HHF), atrial fibrillation and severe hypoglycaemia in patients with type 2 diabetes (T2D) in a real‐world setting.

**Methods:**

All patients with T2D prescribed glucose‐lowering drugs (GLDs) during 2012 to 2015 were identified in nationwide registries in Denmark, Norway and Sweden. Patients were divided into two groups: new users of dapagliflozin and new users of DPP‐4 inhibitors, matched 1:3 by propensity score, calculated by patient characteristics, comorbidities and drug treatment. Cox survival models were used to estimate hazard ratio (HR) per country separately, and a weighted average was calculated.

**Results:**

After matching, a total of 40 908 patients with T2D were identified as new users of dapagliflozin (n = 10 227) or a DPP‐4 inhibitor (n = 30 681). The groups were well balanced at baseline; their mean age was 61 years and 23% had CV disease. The mean follow‐up time was 0.95 years, with a total of 38 760 patient‐years. Dapagliflozin was associated with a lower risk of MACE, HHF and all‐cause mortality compared with DPP‐4 inhibitors: HRs 0.79 (95% confidence interval [CI] 0.67‐0.94), 0.62 (95% CI 0.50‐0.77), and 0.59 (95% CI 0.49‐0.72), respectively. Numerically lower, but non‐significant HRs were observed for myocardial infarction (0.91 [95% CI 0.72‐1.16]), stroke (0.79 [95% CI 0.61‐1.03]) and CV mortality (0.76 [95% CI 0.53‐1.08]) Neutral associations with atrial fibrillation and severe hypoglycaemia were observed.

**Conclusions:**

Dapagliflozin was associated with lower risks of CV events and all‐cause mortality compared with DPP‐4 inhibitors in a real‐world clinical setting and a broad T2D population.

## 1 INTRODUCTION

Despite modern preventive treatment for cardiovascular (CV) complications, patients with type 2 diabetes (T2D) have an increased risk of mortality, heart failure and CV disease.^1^, ^2^ The sodium glucose‐co‐transporter‐2 (SGLT‐2) inhibitors empagliflozin and canagliflozin have recently been shown to be associated with a reduced risk of CV disease and hospitalization for heart failure (HHF) and, in the case of empagliflozin, also a reduced risk of all‐cause mortality, compared with placebo added to existing glucose‐lowering drug (GLD) treatment in patients with T2D with high CV disease risk profile.^3^, ^4^ As part of the CVD‐REAL study programme designed to study the effects of SGLT‐2 inhibitor treatment on CV outcomes in a real‐world setting,^5^, ^6^ the CVD‐REAL Nordic Study, a large multinational observational study of >90 000 patients with T2D, has suggested the presence of SGLT‐2 inhibitor class effects by showing lower risk of CV mortality, major adverse CV events (MACE; non‐fatal myocardial infarction, non‐fatal stroke or CV mortality) and HHF compared with other GLDs^7^; however, the comparator group used in that study,^7^ other GLDs, consisted of almost 50% patients with T2D treated with insulin or sulphonylureas, which have been shown to have increased associated CV risks compared with dipeptidyl peptidase‐4 (DPP‐4) inhibitors in previous observational studies.^8^, ^9^, ^10^, ^11^, ^12^, ^13^, ^14^, ^15^ In addition, it is not fully clear to what extent this could have influenced the risk estimates. Moreover, the comparator group other GLDs does not reflect any particular GLD class but rather the real‐world use of GLDs in patients with T2D, similarly to new use of a SGLT‐2 inhibitor. It is therefore very important to assess CV risks by comparing SGLT‐2 inhibitor treatment with a specific and clinically relevant treatment strategy, for example, DPP‐4 inhibitor therapy. To the best of our knowledge, such an analysis has not been reported before and there are no ongoing CV outcome trials comparing SGLT‐2 with DPP‐4 inhibitor treatment.

The DPP‐4 inhibitors belong to a class of widely used GLDs, which have been shown to be associated with CV safety in several large clinical trials, although concerns about increased heart failure risk have been raised.^16^, ^17^, ^18^ Being a widely used modern oral treatment for T2D, as is SGLT‐2 inhibitor treatment, DPP‐4 inhibitors are a well‐suited comparator for examining the effectiveness of another GLD. Moreover, for second‐ or higher line therapy for patients with T2D, both SGLT‐2 inhibitors and DPP‐4 inhibitors are recommended treatment strategies.^19^

The primary aim of the present study was to investigate if dapagliflozin, the most frequently used SGLT‐2 inhibitor in the Nordic countries,^7^ was associated with risks of MACE, HHF and all‐cause mortality compared with DPP‐4 inhibitors in a broad unselected population with T2D using nationwide data from Denmark, Norway and Sweden. Secondary aims were to study unstable angina, atrial fibrillation and severe hypoglycaemia.

## 2 MATERIALS AND METHODS

### 2.1 Data sources

The three Nordic countries Denmark, Norway and Sweden have comprehensive, nationwide public healthcare systems. All citizens have a unique personal identification number (person‐ID), which is mandatory for all administrative purposes (including any contact with the healthcare system and drug purchases), thus providing a complete full population medical history. In the present study, we used data from Prescribed Drug Registers, the Cause of Death Registers, and the National Patient Registers covering all hospitalizations with discharge diagnoses and all outpatient hospital visits. Individual patient‐level data from the national registers were linked using the person‐ID. The linked databases were managed separately by the Steno Diabetes Centre Copenhagen, Gentofte, Denmark (Danish database) and Statisticon AB, Uppsala, Sweden (Swedish and Norwegian databases). Anonymized data were used and analyses were performed within each separate country database (for detailed country‐specific database information, see Supporting Information Appendix S1 ‐ section 1). The separate studies were approved accordingly by the Danish Data Protection Agency (Datatilsynet, registration number 2015‐41‐4148), the Norwegian Data Protection Agency (registration number 16/00005‐2/GRA) and Regional Committee for Medical and Health Research Ethics South East (registration number 2015/1337/REK), and the Stockholm regional ethics committee (registration number 2013/2206‐31).

### 2.2 Study population

All patients with T2D aged ≥18 years who were new drug users of either dapagliflozin or DPP‐4 inhibitors during the years 2012 to 2015, when dapagliflozin was available in all three countries, were eligible for inclusion (Supporting Information Table S1A). Patients with type 1 diabetes, gestational diabetes and polycystic ovarian syndrome were excluded (Supporting Information Appendix S1 ‐ section 2). A new user date was defined as the date on which the drug class of interest was dispensed, preceded by a 12‐month period with no dispensing of the same drug class. This definition allowed several possible new user dates for a patient within the observation period, both within drug class and between classes. In case multiple new user dates were found, the definition of an index date followed a hierarchical structure, starting with the dapagliflozin new user date if present.

### 2.3 Baseline data

Patient characteristics included age at the date of index drug, sex, index date, date of first dispensing of a registered GLD, and a description of patient frailty (defined as at least 1 hospitalization of ≥3 consecutive days during the year prior to index date).^1^, ^13^, ^14^; detailed definitions are provided in Supporting Information Table S1B. Comorbidities were searched for in all available data prior to and including the index date, with the exceptions of severe hypoglycaemia (within 12 months prior to index date) and cancer (within 5 years prior to index date); detailed definitions are provided in Supporting Information Table S1C. ‘Prior’ medications were defined as any prescription 12 months prior to and including index date. Detailed definitions are provided in Supporting Information Table S1D.

### 2.4 Follow‐up

The primary analyses used an on‐treatment approach. Patients were observed from index date until index drug discontinuation (defined as the first gap of 6 months between filled prescriptions), death, or December 31, 2015. In addition, intention‐to‐treat analyses were performed also including the follow‐up time after index treatment discontinuation.

### 2.5 Definition of outcomes

Outcomes were: MACE, defined by main diagnosis of non‐fatal myocardial infarction or non‐fatal ischaemic/haemorrhagic stroke or CV mortality; HHF, defined by in‐ or outpatient visits with a main diagnosis of heart failure; all‐cause mortality, defined as death from any cause; MACE+, MACE with the addition of unstable angina; and MACE++, MACE with the addition of unstable angina and HHF. Other predefined outcomes were atrial fibrillation and severe hypoglycaemia. Detailed outcome definitions are given in Supporting Information Table S1C.

### 2.6 Propensity score matching

Propensity scores were used to match each patient who initiated dapagliflozin with patients who initiated a DPP‐4 inhibitor (1:3 match, using a caliper of 0.2). The probability of having a new drug initiation of dapagliflozin was estimated using a logistic regression model with patient characteristics, age, time since first GLD initiation, comorbidity, coronary revascularization, frailty, all separate classes of GLDs, CV disease preventive drugs, drugs associated with treatment of heart failure, and date of both index drug and first‐line initiation as independent variables. Detailed information on variables included in the propensity score are given in Supporting Information Tables S2A to C, S3A to C and Appendix S1 ‐ section 3. The matching was performed using the Match function in the R package Matching.^20^

### 2.7 Statistical analysis

Standardized differences of >10% were used to detect significant group imbalance between baseline variables.^21^ The primary analysis was a survival analysis using a Cox proportional hazards model, with time since index date as the underlying time scale. A risk reduction in the dapagliflozin group was considered to be significant when the *P* value was <.05 and the hazard ratio (HR) was <1. Proportional assumptions were tested. Pooled Kaplan–Meier plots from all 3 countries were used for descriptive purposes only.^22^ The primary model used only index drug as a covariate (dapagliflozin vs DPP‐4 inhibitor). All analyses were conducted using R statistical software (R version 3.2.3).^23^

## 3 RESULTS

### 3.1 Unmatched patient characteristics and treatments

**Figure 1:**
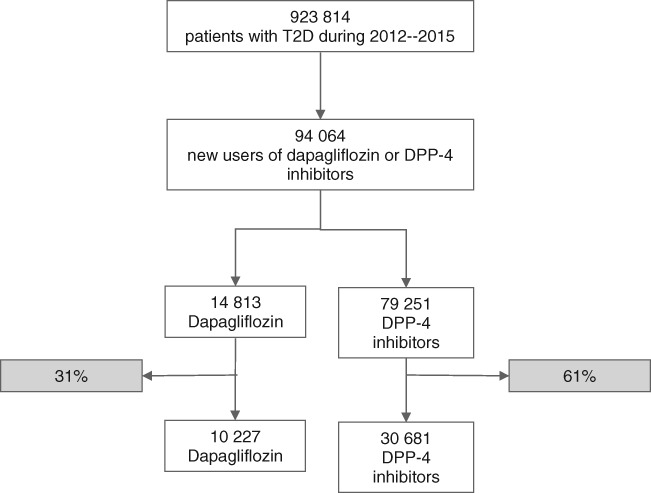
Patient flow charts for dapagliflozin vs DPP‐4 inhibitor groups. Proportion of patients not fulfilling propensity matching 1:3 with 0.2 caliper were excluded and are shown in grey boxes

During the observation period years 2012 to 2015, 94 064 patients with T2D initiated new therapy with dapagliflozin or a DPP‐4 inhibitor (Figure 1). Before matching, patients in the dapagliflozin group were younger, less frequently women, had more microvascular disease and a lower CV burden compared with patients in the DPP‐4 inhibitor group (Supporting Information Table S2). The dapagliflozin and DPP‐4 inhibitor group were similar with respect to CV disease preventive treatment, statins, antihypertensives and low‐dose aspirin.

### 3.2 Propensity score‐matched analyses

After matching, a total of 40 908 patients with T2D could be included as new users of either dapagliflozin (n = 10 227) or a DPP‐4 inhibitor (n = 30 681). The groups were well balanced at baseline: the mean age was 61 years, 40% were women, 23% had CV disease, 15% microvascular disease and 84% had been prescribed CV disease preventive drugs (Table 1). The mean follow‐up time was 0.95 years (dapagliflozin 0.91 years and DPP‐4 inhibitor 0.96 years), with a total of 38 760 patient‐years.

**Table 1:** Baseline patient characteristics of propensity‐matched new users of dapagliflozin vs new users of DPP‐4 inhibitors in a population with T2D

	Dapagliflozin N = 10 227	DPP‐4 inhibitor N = 30 681	Standardized difference^a^
Age, years (s.d.)	61 (11.1)	60.8 (12.4)	0.017
Sex (Female)	4196 (41.0)	12 391 (40.4)	0.011
First GLD, years (s.d.)	6.5 (4.1)	6.5 (4.1)	0.009
CV disease	2356 (23.0)	6970 (22.7)	0.006
Myocardial infarction	730 (7.1)	2183 (7.1)	0.001
Stroke	566 (5.5)	1699 (5.5)	0.000
Unstable angina	286 (2.8)	900 (2.9)	0.007
Heart failure	485 (4.7)	1440 (4.7)	0.002
Atrial fibrillation	879 (8.6)	2549 (8.3)	0.008
Chronic kidney disease	219 (2.1)	626 (2.0)	0.006
Microvascular complications	1497 (14.6)	4449 (14.5)	0.003
Cancer	850 (8.3)	2624 (8.6)	0.007
Metformin	8522 (83.3)	25 705 (83.8)	0.010
Sulphonylurea	2668 (26.1)	7920 (25.8)	0.005
GLP‐1RAs	798 (7.8)	2309 (7.5)	0.008
Thiazolidinediones	148 (1.4)	416 (1.4)	0.006
Insulin	3105 (30.4)	8920 (29.1)	0.023
Short‐acting	1124 (11.0)	3307 (10.8)	0.006
Intermediate‐acting	1504 (14.7)	4358 (14.2)	0.012
Premixed insulin	813 (7.9)	2350 (7.7)	0.009
Long‐acting	1044 (10.2)	3062 (10.0)	0.006
CV disease preventive drugs	8702 (85.1)	26 041 (84.9)	0.005
Low‐dose aspirin	3497 (34.2)	10 434 (34.0)	0.003
Statins	6457 (63.1)	19 405 (63.2)	0.002
Antihypertensives	7483 (73.2)	22 255 (72.5)	0.012
Loop diuretics	1364 (13.3)	4036 (13.2)	0.004
Aldosteron antagonists	441 (4.3)	1303 (4.2)	0.003
Warfarin	527 (5.2)	1530 (5.0)	0.006
Receptor P2Y12 antagonists	471 (4.6)	1351 (4.4)	0.008

Abbreviations: GLP‐1RA, glucagon‐like peptide‐1 receptor agonists; s.d., standard deviation.

All numbers in parenthesis are percentages, unless stated otherwise.

a Standardized difference of >10% (>0.1) is considered to represent a non‐negligible difference.

### 3.3 Cardiovascular disease

**Figure 2:**
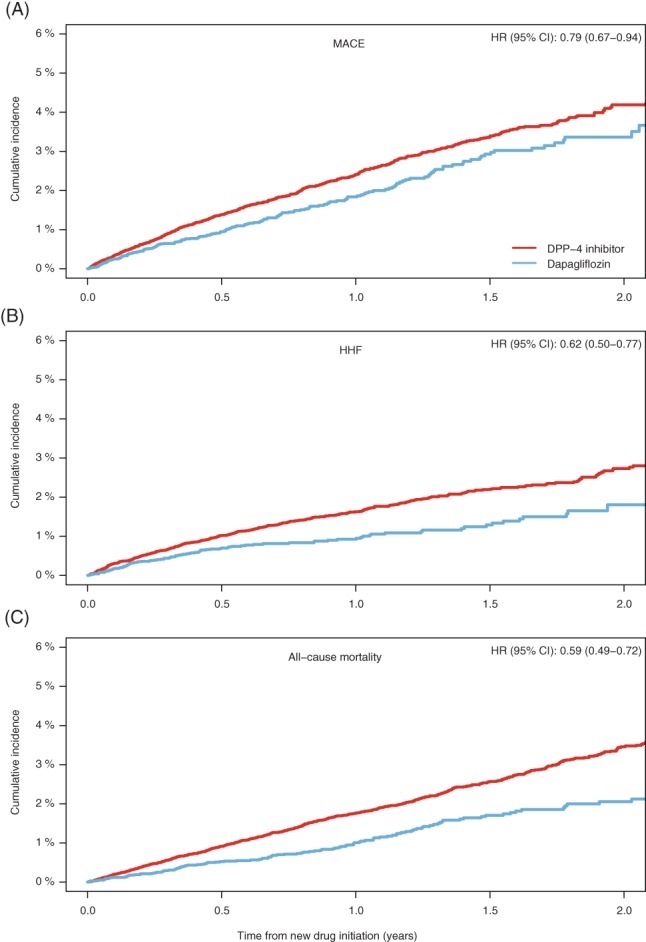
Pooled Kaplan–Meier curves from all 3 countries and HRs comparing propensity score‐matched 1:3 groups of new users of dapagliflozin vs DPP‐4 inhibitor treatment for MACE (A), HHF (B) and all‐cause mortality (C) [Correction added on 18 September 2017 after first online publication: The label in Figure 2 (C) was previously incorrect and has been amended in this version]

The dapagliflozin group was associated with a lower risk of MACE and HHF compared with the DPP‐4 inhibitor group: HRs 0.79 (95% confidence interval [CI] 0.67‐0.94) and 0.62 (0.50‐0.77), respectively (Table 2 and Figure 2). The risk of non‐fatal myocardial infarction, non‐fatal stroke and CV mortality was non‐significantly lower in the dapagliflozin group: HRs 0.91 (95% CI 0.72‐1.16), 0.79 (95% CI 0.61‐1.03), and 0.76 (95% CI 0.53‐1.08), respectively. Lower HRs for MACE+ and MACE++ in the dapagliflozin group were observed: HRs 0.81 (95% CI 0.69‐0.94) and 0.75 (95% CI 0.66‐0.86), respectively. No associations were observed with unstable angina.

**Table 2:** Weighted means of HRs in Denmark, Norway and Sweden for new users of dapagliflozin vs new users of DPP‐4 inhibitors

	Dapagliflozin N = 10 227		DPP‐4 inhibitor N = 30 681		Weighted average estimates N = 40 908		
	No. events	Events/100 patient‐years	No. events	Events/100 patient‐years	HR	95% CI	*P*
MACE	177	1.86	695	2.34	0.79	(0.67‐0.94)	0.006
Non‐fatal myocardial infarction	87	0.91	304	1.02	0.91	(0.72‐1.16)	0.445
Non‐fatal stroke	69	0.72	270	0.90	0.79	(0.61‐1.03)	0.086
Cardiovascular mortality	38	0.40	160	0.53	0.76	(0.53‐1.08)	0.122
HHF	95	0.99	467	1.57	0.62	(0.50‐0.77)	<0.001
MACE+	202	2.12	779	2.63	0.81	(0.69‐0.94)	0.007
Unstable angina	37	0.39	107	0.36	1.09	(0.75‐1.59)	0.655
MACE++	285	3.01	1164	3.96	0.75	(0.66‐0.86)	<0.001
All‐cause mortality	120	1.03	644	1.75	0.59	(0.49‐0.72)	<0.001
Atrial fibrillation	140	1.47	469	1.58	0.92	(0.76‐1.12)	0.414
Severe hypoglycaemia	91	0.95	300	1.01	0.94	(0.74‐1.19)	0.618

The groups were matched 1:3 using propensity scores based on age, sex, frailty (≥3 days in hospital within 1 year prior to index) comorbidity and treatment.

### 3.4 Other outcomes

There was a lower HR for all‐cause mortality (0.59 [95% CI 0.49‐0.72]) in the dapagliflozin group compared with the DPP‐4 inhibitor group (Table 2 and Figure 2). The dapagliflozin group showed neutral associations for atrial fibrillation and severe hypoglycaemia compared with DPP‐4 inhibitor: HRs 0.92 (95% CI 0.76‐1.12) and 0.94 (95% CI 0.74‐1.19), respectively.

### 3.5 Sensitivity analyses

When including time after treatment discontinuation (eg, intention‐to‐treat), the analysis showed similar risk associations between the dapagliflozin group and the DPP‐4 inhibitor group (Supporting Information Table S4). Risk estimation for heart failure registered in‐hospital only (ie, excluding outpatient events) remained unchanged compared with HHF, including both in‐ and outpatient events (Supporting Information Table S4).

## 4 DISCUSSION

In this large population of >40 000 patients with T2D from 3 countries, covering ~20 million inhabitants, new use of dapagliflozin was associated with 21% lower risk of MACE and 38% lower risk of HHF compared with new use of DPP‐4 inhibitors. In addition, a 41% lower all‐cause mortality risk was observed. The MACE components, myocardial infarction, stroke and CV mortality, did separately show lower, but non‐significant differences. Extended outcome combinations of MACE, adding unstable angina and HHF, respectively, did not change the lower risk associations with dapagliflozin compared with DPP‐4 inhibitors. Neutral associations were found with severe hypoglycaemia and atrial fibrillation.

Similar to our reported 21% (HR 0.79 [95% CI 0.67‐0.94]) associated lower risk of MACE, a recent meta‐analysis of dapagliflozin treatment in patients with 30% established CV disease at baseline showed a numerically 23% (HR 0.77 [95% CI 0.54‐1.10]) lower risk of MACE compared with placebo.^24^ Furthermore, the numerical risk reductions for MACE+ reported by the same meta‐analysis also showed similarities with our results: HRs 0.79 (95% CI 0.58‐1.07) and 0.81 (95% CI 0.69‐0.94) respectively.^24^ Despite not being significant, these meta‐analyses on MACE outcomes, indirectly provide clinical trial data support to our real‐world results.

The EMPA‐REG OUTCOME trial showed that empagliflozin reduced the risk of MACE, HHF, and all‐cause mortality compared with placebo by 14%, 35% and 32%, respectively, also similar to canagliflozin in the CANVAS trial.^3^, ^4^ This was similar to our findings of 21%, 38% and 41%, respectively, but an important difference was the substantially lower CV risk profile at baseline: in the present population there was 23% established CV disease compared with 99% and 72%, respectively, in the EMPA‐REG OUTCOME and CANVAS trials. This may indicate that the risk‐lowering effects reported for empagliflozin and canagliflozin also extend to dapagliflozin, and to a T2D patient population with a substantially lower CV risk profile at baseline.

Heart failure is a both common and frequently underdiagnosed complication in T2D,^25^, ^26^ increasing mortality and CV risks,^27^ which emphasizes the importance of its prevention and treatment. We show that dapagliflozin treatment, compared with DPP‐4 inhibitor treatment, is associated with significantly lower risks of HHF in patients with a broader CV risk profile than in clinical trials. As evidence‐based treatment of heart failure in T2D is currently lacking,^28^ these new findings might be of particular clinical importance while waiting for results from ongoing randomized mechanistic and outcome trials of dapagliflozin (Clinicaltrials.gov: NCT03030235, NCT02653482 and NCT03036124).

Interestingly, both atrial fibrillation and severe hypoglycaemia showed no associations between the two treatment groups in the present work. The risk of atrial fibrillation has not, to our knowledge, been reported to show associations with either SGLT‐2 or DPP‐4 inhibitors, and should thus be expected to be neutral. In addition, it is known that both dapagliflozin and DPP‐4 inhibitors have low and similar risks of hypoglycaemia.^29^ Hence, these expected neutral associations suggest that the treatment groups were well balanced at baseline regarding disease burden, and including unknown confounders.

In contrast to the EMPA‐REG OUTCOME trial, reporting non‐significant higher stroke risk (HR 1.24 [95% CI 0.92‐1.67]), we report a lower risk association for stroke (HR 0.79 [95% CI 0.61‐1.03]) with dapagliflozin compared with DPP‐4 inhibitor treatment. This finding is of particular interest because it is more in line with the CANVAS trial results (HR 0.90 [05% CI 0.711‐1.15]) and considers benefit in stroke rates that could be expected by a mild reduction in blood pressure. The seemingly discrepant findings between our results and those of the EMPA‐REG OUTCOME trial could be attributable to chance and/or presence of a potential unknown confounding factor at baseline; however, the virtually identical baseline variables, similar risk estimates for other outcomes compared with the EMPA‐REG OUTCOME and CANVAS trials^3^, ^4^ and absent atrial fibrillation and hypoglycaemia associations all support our results.

Previous observational multi‐country studies have recently shown that parts of the EMPA‐REG OUTCOME and CANVAS trial results do translate to the SGLT‐2 inhibitor class, and into a real‐world setting with patients with T2D having broader CV risk profiles^3^, ^4^, ^6^, ^7^, ^30^; however, by using a wider range of outcomes, the present paper further adds to this knowledge base by showing that a specific SGLT‐2 inhibitor, dapagliflozin, is associated with lower CV risk in a real‐world setting compared with a widely used GLD class, DPP‐4 inhibitors. Observational effectiveness studies cannot replace randomized clinical trials, but might prove to be an important complement to these, translating results to a broader and more generalized patient population in a real‐world setting.^31^ While awaiting more complete evidence, observational comparative effectiveness studies may increase the understanding of the SGLT‐2 inhibitor treatment outcome effects; however, confounding, particularly confounding by indication, cannot be fully excluded in observational studies, and the large ongoing prospective trial DECLARE‐TIMI 58 (clinicaltrials.gov, NCT01730534), including >17 000 patients with both low and high CV risk, will further elucidate dapagliflozin‐specific findings.

The strengths of the present study include its population‐based, nationwide and unselected real‐world design, which provides high external validity and a large enough population to allow country‐wise propensity score‐matched analyses to be performed. The results were consistent across all 3 countries and across several subgroup analyses. In addition, national registers with full coverage for hospitalizations, filled drug prescriptions and cause of death were used in 3 countries with established and complete public healthcare systems. Because diagnostic accuracy can be challenging for HHF in registries, it is reassuring that CV diagnoses in Denmark, Norway and Sweden have high validity.^32^, ^33^, ^34^, ^35^, ^36^ Anticipated neutral associations with atrial fibrillation and severe hypoglycaemia confirms the balanced risk profile at baseline for the dapagliflozin and DPP‐4 inhibitor groups.

Limitations of the study include the fact that the results are only representative of patients who have been initiated on SGLT‐2 inhibitor treatment or who are similar with regard to available clinical variables and, therefore, cannot be extended to all patients with T2D. The present work has no information on laboratory measurements, lifestyle variables, primary healthcare data, or socio‐economic data, and consequently there may be remaining confounding factors. The close matching on a large number of essential variables ensures that some confounding factors are controlled for, but even propensity score matching does not remedy all confounding; for example, residual confounding by indication. Further, there was no information on diabetes duration, therefore, we used a proxy for time since diagnosis by matching for age at index date, time since first registered GLD treatment and classes of GLD at baseline. Because dapagliflozin has only been on the market since 2012 the mean follow‐up was short, ~1 year; however, in the EMPA‐REG OUTCOME study, similar early effects to those of the present study have been observed, suggesting that the 1‐year mean follow‐up time might be sufficient. We did not examine safety, but recent reports have not identified any new safety signals with dapagliflozin.^37^, ^38^ For Norway and Sweden, we had no information on emigration, which could result in loss to follow‐up. No information on immigration was available and some patients might have less comprehensive disease history; however, the on‐treatment analyses used should minimize the effects of patients emigrating because they would be classified as discontinuing treatment. Furthermore, the results were consistent with those of Denmark, where migration information was included.

In conclusion, dapagliflozin, when compared with DPP‐4 inhibitor treatment, was associated with a lower risk of MACE, heart failure and all‐cause mortality in a real‐world T2D population, where 23% had previously established CV disease. A large ongoing prospective dapagliflozin trial will further elucidate these findings.

## ORCID

*Jan W. Eriksson* http://orcid.org/0000-0002-2639-9481

*Johan Bodegard* http://orcid.org/0000-0001-5423-3967

## Supporting information


**Appendix S1** Data sources.

**Table S1A.** Definitions of glucose lowering drugs.
**Table S1B.** Definitions of patient characteristics.
**Table S1C.** International Classification of Diseases [ICD] code 8/9/10 diagnoses and Classification of Surgical Procedures NOMESCO (Nordiska medicinalstatistiska kommittén) codes used to define comorbidities and treatments.
**Table S1D.** Prior medications using the ATC (Anatomical Therapeutic Chemical) codes.
**Table S2.** Baseline patient characteristics of unmatched type 2 diabetes patients being new users of dapagliflozin versus dipeptidyl peptidase‐4 inhibitor (DPP‐4i).
**Table S3.** Baseline of propensity matched 1:3 type 2 diabetes patients being new users of dapagliflozin versus dipeptidyl peptidase‐4 inhibitor (DPP‐4i).
**Table S4.** Weighted means of hazard ratios (HRs) in Denmark, Norway and Sweden for new users of dapagliflozin versus dipeptidyl peptidase‐4 inhibitor (DPP‐4i) including the follow‐up time after index treatment discontinuation (intention to treat) and separate analysis on inpatient hospitalization for heart failure. The groups were matched 1:3 using propensity scores based on age, sex, frailty (three or more days in hospital within one year prior to index) comorbidity and treatment
