# Supplementary material for: Dapagliflozin is associated with lower risk of cardiovascular events and all‐cause mortality in people with type 2 diabetes (CVD‐REAL Nordic) when compared with dipeptidyl peptidase‐4 inhibitor therapy: A multinational observational study
Source: Diabetes Obes Metab. 2017 Sep 8;20(2):344–51. doi: 10.1111/dom.13077 (PMC5811811; doi:10.1111/dom.13077)
Supplement: Supplementary file 1 — Appendix S1 Data sources. [file DOM-20-344-s001.docx]

## Data Sources

Data from nationwide cohort studies including T2D patients from Sweden, Denmark and Norway was included in these analyses to obtain a homogenous study population of appropriate size. A short description of the respective databases is provided below.

**Denmark:** The DAFFODIL study database includes T2D patient information from three linked national Danish registries with full coverage of the Danish population: the Prescribed Drug Register (1990 to 2015) covering all filled drug prescriptions using ATC codes; the Cause of Death Register (1952– to 2015); and the National Patient Register covering all open patient clinic visit diagnoses for 2000 to 2015 and all hospital discharge diagnoses for the years 1980 to 2015, and discharge diagnoses and The Cause of Death Register was updated until 31^st^ of December 2014. Diagnoses are recorded according to the ICD-system. All three registers are held by the Statistics Denmark. Data from Statistics Denmark were made available following an application to Statistics Denmark (<http://www.dst.dk/en/TilSalg/Forskningsservice>). Data from the three registers were merged by Statistics Denmark by using unique personal identification numbers(1, 2) and analyses were performed via a secure VPN connection. Due to the Danish Act of processing of personal data, future interested researchers have to perform the above-mentioned steps to obtain access to the data.

**Norway:** The DAPHNE study database includes T2D patient information from three linked national Norwegian registries with full coverage of the Norwegian population: the Norwegian Prescription Database (July 2004 to December 2015) covering all filled drug prescriptions using ATC codes; the Norwegian Cause of Death Registry (1958 to 2015)(3); and the Norwegian Patient Register covering all open patient clinic visit diagnoses and all hospital discharge diagnoses for the years 2008 to 2015. The Cause of Death Registry was updated until 31^st^ of December 2015. Diagnoses are recorded according to the ICD-system. Data linkage is performed by the Norwegian Institute of Public Health.

**Sweden:** The DAISY study database (4) includes T2D patient information from three linked national Swedish registries with full coverage of the Swedish population: the Prescribed Drug Register (July 2005 to December 2015) covering all filled drug prescriptions using the Anatomical Therapeutic Chemical (ATC) codes; the Cause of Death Registry (1961 to 2015); and the National Patient Registry covering all open patient clinic visit diagnoses for 2001 to 2015 and all hospital discharge diagnoses for the years 1987–2015. Diagnoses are recorded according to the ICD-system and has been shown to be of high validity. All three registers are held by the Swedish National Board of Health and Welfare (NBHW), who also performs the data linkage by using unique personal identification numbers.(5)

# Study population

## Type 2 diabetes population

In Sweden and Norway, a type 2 diabetic patient is defined as all patients prescribed with any blood glucose lowering drug in the drug register excluding patients with type 1 diabetes and patients with gestational diabetes or polycystic ovary syndrome.

**Type 1 diabetes** is defined as:

- Patients with a type 1 diabetes diagnose (E10) and as long as insulin is used as monotherapy. At the first start of a non-insulin antidiabetic drug, a patient is considered as a type 2 diabetic patient from that date.

OR

- Age <30 at start of insulin medication

OR

- Age <15 at start of diabetes medication

**Gestational diabetes** is defined as:

- In or outpatient visit with ICD10: O24.4 will create a wash out window of 1 year. The first dispense of blood glucose lowering will classify a patient as a type 2 diabetic patient (unless criteria for type 1 is fulfilled)

**Polycystic ovary syndrome (PCOS)** is defined as:

In or outpatient visit with ICD10: O28.2 or combination treatment with ATC-code G03GB02 and metformin. Metformin therapy after 45 years of age, or treatment with any other antidiabetic drug will classify the patient as a type 2 diabetic patient from that date.

**Danish diabetes patients are defined from:**

- The Danish National Diabetes Register (NDR) using only the non-blood glucose criteria (period 1995--2012). This register contains one record per person.
- The National Patient Register (NPR) using the contact records with a diabetes diagnosis (period 1977--2015).
- The Danish Adult Diabetes Database (DADD) using the annual
    reports (period 2005--2015)
- The Register of Medicinal Product Statistics (RMPS) using diabetes drug purchase (period 1995—2015).
- The eye screening database with records of all retina screening visits (2009--2015).

Persons found in any of these registers are taken to be a diabetes patient, and the date of diagnosis to be the earliest recorded.
The population of T2D patients is formed by excluding persons assumed to be T1D patients:

- any purchase of antidiabetic drug before age 15
- any purchase of insulin before age 30
- recording of diabetes type as T1D in the DADD (most frequent recording)

Furthermore, women with a diagnosis of gestational diabetes in the NPR are not included as diabetes patients in the first year after the diagnosis of gestational diabetes, and women with a recorded diagnosis of PCOS are not included as diabetes patients on the basis of metformin purchase.

# Propensity score matching

The probability of having a new drug initiation of dapagliflozin will be estimated using a logistic regression model with group (dapagliflozin = 1 and DPP-4i = 0) as the dependent variable and

age, gender, diabetes duration, history of myocardial infarction, unstable angina, angina pectoris, coronary revascularization (PCI and CABG), heart failure, atrial fibrillation, stroke, transitory ischemic attack, peripheral artery disease, major organ specific bleeding, bariatric surgery, microvascular complications, severe hypoglycemia, lower limb amputations, chronic obstructive pulmonary disease, kidney disease, cancer, frailty (defined as 3 or more days of hospitalization during the year prior to index date), all separate GLDs, drugs to prevent or treat CVD (angiotensin-converting-enzyme inhibitors, angiotensin receptor blockers, beta-blockers, low-/high ceiling diuretics, aldosterone antagonists, warfarin, digitoxin/digoxin, flekanide, amiodarone, statins, low dose acetylsalicylic acid, receptor P2Y_12_ antagonists, direct factor Xa inhibitor (novel oral anti-coagulants), other antiplatelets, dihydropyridines, non-dihydropyridines, low ceiling diuretics, corticosteroids, weight loss drugs, different classes of diabetes medications, frailty and calendar year of both index date and first line initiation.

The propensity score from the model will then be used to match each dapagliflozin patient with three DPP-4i patients using an automated balance optimization method using the function Match in package Matching in R using a caliper of 0.2.

**References**

1. Pedersen CB. The Danish Civil Registration System. Scand J Public Health. 2011;39(7 Suppl):22-5.

2. Schmidt M, Pedersen L, Sorensen HT. The Danish Civil Registration System as a tool in epidemiology. Eur J Epidemiol. 2014;29(8):541-9.

3. Norwegian Cause of Death Registry [Available from: <https://www.fhi.no/en/hn/health-registries/cause-of-death-registry/>.

4. Norhammar A, Bodegård J, Nyström T, Thuresson M, Eriksson JW, Nathanson D. Incidence, prevalence and mortality of type 2 diabetes requiring glucose-lowering treatment, and associated risks of cardiovascular complications: a nationwide study in Sweden, 2006-2013. Diabetologia. 2016;59(8):1692-701.

5. Ludvigsson JF, Otterblad-Olausson P, Pettersson BU, Ekbom A. The Swedish personal identity number: possibilities and pitfalls in healthcare and medical research. Eur J Epidemiol. 2009;24(11):659-67.
