# Supplementary material for: Dapagliflozin is associated with lower risk of cardiovascular events and all‐cause mortality in people with type 2 diabetes (CVD‐REAL Nordic) when compared with dipeptidyl peptidase‐4 inhibitor therapy: A multinational observational study
Source: Diabetes Obes Metab. 2017 Sep 8;20(2):344–51. doi: 10.1111/dom.13077 (PMC5811811; doi:10.1111/dom.13077)
Supplement: Supplementary file 2 — Table S1A. Definitions of glucose lowering drugs. Table S1B. Definitions of patient characteristics. Table S1C. International Classification of Diseases [ICD] code 8/9/10 diagnoses and Classification of Surgical Procedures NOMESCO (Nordiska medicinalstatistiska kommittén) codes used to define comorbidities and treatments. Table S1D. Prior medications using the ATC (Anatomical Therapeutic Chemical) codes. Table S2. Baseline patient characteristics of unmatched type 2 diabetes patients being new users of dapagliflozin versus dipeptidyl peptidase‐4 inhibitor (DPP‐4i). Table S3. Baseline of propensity matched 1:3 type 2 diabetes patients being new users of dapagliflozin versus dipeptidyl peptidase‐4 inhibitor (DPP‐4i). Table S4. Weighted means of hazard ratios (HRs) in Denmark, Norway and Sweden for new users of dapagliflozin versus dipeptidyl peptidase‐4 inhibitor (DPP‐4i) including the follow‐up time after index treatment discontinuation (intention to treat) and separate analysis on inpatient hospitalization for heart failure. The groups were matched 1:3 using propensity scores based on age, sex, frailty (three or more days in hospital within one year prior to index) comorbidity and treatment [file DOM-20-344-s002.docx]

**Supplemental Table 1a** — Definitions of glucose lowering drugs

| **Drug class** | **ATC code** |
| --- | --- |
| SGLT-2 inhibitors | A10BK01, A10BK02, A10BK03 or A10BD15, A10BD16, A10BD20 (in combination) |
| Metformin | A10BA, or A10BD (in combination) |
| Sulfonylurea | A10BB |
| DPP-4 inhibitors | A10BH, or A10BD07, A10BD08, A10BD10 (in combination) |
| GLP-1RA | A10BJ01, A10BJ02, A10BJ03, A10BJ05 |
| Metiglinides | A10BX02, A10BX03 or A10BD03, A10BD04, A10BD05 (in combination) |
| Glitazones | A10BG |
| Acarbose | A10BF |
| Insulins |  |
| *Short-acting* | A10AB |
| *Intermediate-acting (isophane)* | A10AC |
| *Premixed insulin* | A10AD |
| *Long-acting* | A10AE |

SGLT-2, sodium–glucose cotransporter 2; DPP, dipeptidyl peptidase; GLP1-RA, glucagon-like peptide-1 receptor agonist

**Supplemental Table 1b** — Definitions of patient characteristics

| **Variable** | **Definition** |
| --- | --- |
| **Sex** |  |
| **Age** |  |
| **Year of first diabetes medication** | Calendar year of the date when the first dispense of any A10 medication was found |
| **Time from first diabetes medication to index date** |  |
| **Year of Index date** |  |
| **Frailty** | At least one hospitalization of at least 3 consecutive days during the year prior to index |

**Supplemental Table 1c** — International Classification of Diseases [ICD] code 8/9/10 diagnoses and Classification of Surgical Procedures NOMESCO (Nordiska medicinalstatistiska kommittén) codes used to define comorbidities and treatments

| **Disease** | **ICD-8** | **ICD-9** | **ICD-10** | **Surgical code** |
| --- | --- | --- | --- | --- |
| **CVD** |  |  |  |  |
| **Myocardial infarction** | 410.9, 410.99 | 410 | I21-I22 |  |
| **CABG** |  | 414.02-07, V45.81-82 |  | FNA-FNE |
| **PCI with stent** |  |  |  | FNG |
| **Unstable angina** |  | 411 | I20.0 |  |
| **Angina pectoris** | 4193, 4139 | 413, 414.0 | I20.1, I20.8, I20.9 |  |
| **Heart failure** | 425.99, 427.09–427.19, 427.99, 428.99 | 428 | I50 |  |
| **Atrial fibrillation** | 427.93, 427.94 | 427.3 | I48 |  |
| **Stroke** |  | 430-438, V125 | I60-I66, G45 |  |
| *Hemorrhagic* | 43000-43099, 43100, 43108–43190, 43198-43199 | 430-432 | I60-I62 |  |
| *Ischemic* | 43200–43299, 43309–43399, 43409-43499 | 433-434, 436 | I63-I64 |  |
| *Transitory ischemic attack* | 43509-43599 | V12.5, 435 | G45 |  |
| **Peripheral artery disease** | 440.20–440.30 | 440/441/444 | I70-I79 |  |
| **Major organ specific bleeding** | 7841,7847 | 578.0/578.1 | D629, I60, I61, I62, I850, K226, K250, K252, K254, K256, K260, K262, K264, K266, K270, K272, K274, K276, K280, K282, K284, K286, K290, K625, K920, K921, K922 |  |
| **Bariatric surgery** |  |  |  | JDF10, JDF11, JDF20, JDF21 |
| **Chronic kidney disease** | 581.00–582.09 | 585 | N18 |  |
| **Dialysis** |  |  | Z49 | JAK10, TJA20, TJA33, DJ008, DR015-24, QF006 |
| **Microvascular complications** |  |  |  |  |
| *Diabetic mono-/polyneuropathy* | 357,355,356,357 | 354.0-355.9, 357.2, 250F | G99.0, G59.0, G63.2, E10.4, E11.4, E12.4, E13.4, E14.4 |  |
| *Diabetic eye complications* | 250.02,3770,3771,3779,4569, 374,3789 | 362, 366.41, 365.44, 362.07, 250E | H28.0, H35.8, H36.0, E10.3, E11.3, E12.3, E13.3, E14.3 | CKC12, CKD65 |
| *Diabetic foot/Peripheral angiopathy* | 250 | 713.5, 250.7, 250G | E11.6B, M14.2, M14.6, M90.8, L98.4, E10.5, E11.5, E12.5, E13.5, E14.5 | QDGX10 |
| *Diabetic kidney disease* | 583 | 583.81, 250D | N08.3, E10.2, E11.2, E12.2, E13.2, E14.2 |  |
| *Diabetes with several-/unspecified complications* |  | 250H-X | E11.6, E10.6, E13.6, E14.6, E10.7, E11.7, E12.7, E13.7, E14.7, E10.8, E11.8, E12.0, E12.8, E13.8, E14.8 |  |
| **Severe hypoglycemia** | 251,9623 | 251,9623 | E10.0, E11.0, E12.0, E13.0, E14.0, E11.6A, E16.0-2 |  |
| **Keto-/lactate acidosis** |  | 249.1/250 | E10.1, E11.1, E12.1, E13.1, E14.1, E87.2 |  |
| **Cancer** | 140.0–204.4 | 140-239 | C00-C99 |  |
| **COPD** | 491.00–492.00 | 491 | J44 |  |
| **Lower limb amputations** |  |  |  | NGQ, NHQ |

CABG, Coronary bypass surgery. PCI, Percutaneous coronary intervention.

**Supplemental Table 1d** — Prior medications using the ATC (Anatomical Therapeutic Chemical) codes

| Drug | ATC code |
| --- | --- |
| **Blood glucose lowering drugs** |  |
| Metformin | A10BA |
| Sulfonylureas | A10BB |
| DPP-4 inhibitors | A10BH |
| SGLT-2 inhibitors | A10BX09, A10BK02, A10BX12 or A10BD15, A10BD16, A10BD20 |
| GLP-1RA | A10BX04, A10BJ02, A10BX10, A10BX14 |
| Metiglinides | A10BX02 and A10BX03 |
| Glitazones | A10BG |
| Acarbose | A10BF |
| Insulins | A10 |
| *Short-acting* | A10AB |
| *Intermediate-acting (isophane)* | A10AC |
| *Premixed insulin* | A10AD |
| *Long-acting* | A10AE |
| Combination Metformin+DPP-4 inhibitors | A10BD07, A10BD08, A10BD10 |
| Combination Metformin+Metiglinid | A10BD03, A10BD04, A10BD05 |
| Combination Metformin+SGLT-2 inhibitors | A10BD15, A10BD16, A10BD20 |
| **CVD risk treatment** |  |
| Low dose acetylic salicylic acid | B01AC06 |
| Statins | C10AA |
| Antihypertensives |  |
| *ACE inhibitors* | C09A, C09B |
| *ARB* | C09C, C09D (exclude C09DX04) |
| *Dihydropyridines (calcium channel blockers)* | C08C |
| *Low ceiling diuretics (thiazides)* | C03A |
| *Beta blockers* | C07 |
| Non-hydropyridines (calcium channel blockers) | C08D |
| High ceiling diuretics (loop-diuretics) | C03C |
| Aldosterone antagonists | C03DA |
| Neprilysine inhibitor | C09DX04 |
| Digitoxin | C01AA04 |
| Digoxin | C01AA05 |
| Flekanide | C01BC04 |
| Amiodarone | C01BD01 |
| Warfarin | B01AA03 |
| Receptor P2Y12 antagonists | B01AC04, B01AC22, B01AC24 |
| Direct factor Xa inhibitors | B01AF |
| Direct thrombin inhibitor | B01AE07 |
| Other antiplatelets | B01AC07, B01AC09, B01AC11,  B01AC13, B01AC16, B01AC17, B01AC21 |
| Corticosteroids | H02 |
| Weight loss drugs | A08A |

SGLT-2, sodium–glucose cotransporter 2; DPP, dipeptidyl peptidase; GLP1-RA, glucagon-like peptide-1 receptor agonist; ACE, angiotensin-converting enzyme; ARB, angiotensin receptor blocker.

**Supplemental Table 2** — Baseline patient characteristics of **unmatched** type 2 diabetes patients being new users of dapagliflozin versus dipeptidyl peptidase-4 inhibitor (DPP-4i)

|  | Dapagliflozin | DPP-4i | Standardized difference (%) |
| --- | --- | --- | --- |
| No. of patients | 14813 | 79251 |  |
| Age, years (SD) | 59.7 (10.9) | 64.9 (12.5) | 0.438 |
| Gender (Female) | 6,018 (40.6) | 31,607 (39.9) | 0.012 |
| First GLD, years | 7.3 (4.1) | 6.1 (4.2) | 0.302 |
| Index year |  |  |  |
| 2012 | 15 (0.1) | 577 (0.7) | 0.092 |
| 2013 | 2,328 (15.7) | 18,250 (23.0) | 0.156 |
| 2014 | 6,061 (40.9) | 27,222 (34.3) | 0.110 |
| 2015 | 6,409 (43.3) | 33,202 (41.9) | 0.023 |
| First GLD year |  |  |  |
| 1995 | 264 (1.8) | 934 (1.2) | 0.040 |
| 1996 | 85 (0.6) | 345 (0.4) | 0.016 |
| 1997 | 101 (0.7) | 368 (0.5) | 0.023 |
| 1998 | 121 (0.8) | 471 (0.6) | 0.021 |
| 1999 | 151 (1.0) | 533 (0.7) | 0.030 |
| 2000 | 184 (1.2) | 619 (0.8) | 0.036 |
| 2001 | 197 (1.3) | 731 (0.9) | 0.031 |
| 2002 | 203 (1.4) | 845 (1.1) | 0.022 |
| 2003 | 251 (1.7) | 1,071 (1.4) | 0.022 |
| 2004 | 1,951 (13.2) | 7,450 (9.4) | 0.095 |
| 2005 | 3,079 (20.8) | 12,699 (16.0) | 0.099 |
| 2006 | 1,035 (7.0) | 4,719 (6.0) | 0.034 |
| 2007 | 927 (6.3) | 4,795 (6.1) | 0.007 |
| 2008 | 949 (6.4) | 5,286 (6.7) | 0.009 |
| 2009 | 904 (6.1) | 5,373 (6.8) | 0.023 |
| 2010 | 884 (6.0) | 5,696 (7.2) | 0.041 |
| 2011 | 821 (5.5) | 5,738 (7.2) | 0.058 |
| 2012 | 727 (4.9) | 5,489 (6.9) | 0.072 |
| 2013 | 770 (5.2) | 6,245 (7.9) | 0.092 |
| 2014 | 722 (4.9) | 5,600 (7.1) | 0.078 |
| 2015 | 487 (3.3) | 4,244 (5.4) | 0.086 |
| Frailty | 2,544 (17.2) | 17,874 (22.6) | 0.112 |
| Cardiovascular disease | 3,429 (23.1) | 23,553 (29.7) | 0.124 |
| Myocardial infarction | 1,017 (6.9) | 7,426 (9.4) | 0.077 |
| Coronary artery bypass grafting | 263 (1.8) | 1,682 (2.1) | 0.021 |
| Percutaneous Coronay intervention | 781 (5.3) | 4,493 (5.7) | 0.014 |
| Unstable angina | 559 (3.8) | 3,301 (4.2) | 0.017 |
| Angina pectoris | 1,462 (9.9) | 8,604 (10.9) | 0.027 |
| Heart failure | 688 (4.6) | 6,353 (8.0) | 0.118 |
| Atrial fibrillation | 967 (6.5) | 8,120 (10.2) | 0.114 |
| Stroke | 893 (6.0) | 7,130 (9.0) | 0.095 |
| *Hemorrhagic* | 93 (0.6) | 884 (1.1) | 0.045 |
| *Ischemic* | 573 (3.9) | 4,697 (5.9) | 0.081 |
| *Transitory ischemic attack* | 334 (2.3) | 2,474 (3.1) | 0.045 |
| Peripheral artery disease | 732 (4.9) | 5,137 (6.5) | 0.055 |
| Major organ specific bleeding | 457 (3.1) | 3,195 (4.0) | 0.043 |
| Bariatric surgery | 63 (0.4) | 169 (0.2) | 0.029 |
| Chronic kidney disease | 118 (0.8) | 4,077 (5.1) | 0.242 |
| Dialysis | 11 (0.1) | 363 (0.5) | 0.070 |
| Microvascular complications | 3,952 (26.7) | 15,798 (19.9) | 0.129 |
| *Neuropathy* | 576 (3.9) | 1,897 (2.4) | 0.067 |
| *Eye complications* | 1,475 (10.0) | 5,365 (6.8) | 0.092 |
| *Peripheral angiopathy* | 266 (1.8) | 1,232 (1.6) | 0.015 |
| *Kidney disease* | 362 (2.4) | 2,162 (2.7) | 0.015 |
| *Several-/unspecified complications* | 2,399 (16.2) | 9,373 (11.8) | 0.101 |
| Severe hypoglycemia | 326 (2.2) | 1,556 (2.0) | 0.013 |
| Keto-/lactate acidosis | 110 (0.7) | 456 (0.6) | 0.017 |
| Cancer | 840 (5.7) | 6,914 (8.7) | 0.100 |
| COPD | 554 (3.7) | 4,106 (5.2) | 0.059 |
| Lower limb amputations | 49 (0.3) | 353 (0.4) | 0.015 |
| Metformin | 12,474 (84.2) | 64,811 (81.8) | 0.053 |
| Sulphonylurea | 3,661 (24.7) | 22,237 (28.1) | 0.062 |
| GLP-1RA | 4,742 (32.0) | 2,352 (3.0) | 0.603 |
| Metiglinides | 264 (1.8) | 1,405 (1.8) | 0.001 |
| Thiazolidinediones | 280 (1.9) | 640 (0.8) | 0.072 |
| Acarbose | 95 (0.6) | 277 (0.3) | 0.032 |
| Insulin | 5,579 (37.7) | 14,282 (18.0) | 0.354 |
| Short-acting | 2,195 (14.8) | 4,780 (6.0) | 0.223 |
| Intermediate-acting | 2,424 (16.4) | 7,231 (9.1) | 0.171 |
| Premixed insulin | 1,338 (9.0) | 3,857 (4.9) | 0.128 |
| Long-acting | 2,364 (16.0) | 4,222 (5.3) | 0.266 |
| Metformin+Metiglinides | 18 (0.1) | 20 (0.0) | 0.026 |
| CVD risk treatment | 12,905 (87.1) | 68,740 (86.7) | 0.009 |
| Low dose aspirin | 5,248 (35.4) | 28,738 (36.3) | 0.014 |
| Statins | 9,869 (66.6) | 51,375 (64.8) | 0.031 |
| Antihypertensives | 11,115 (75.0) | 59,159 (74.6) | 0.007 |
| ACE inhibitors | 4,725 (31.9) | 26,170 (33.0) | 0.020 |
| ARB | 5,749 (38.8) | 27,045 (34.1) | 0.079 |
| Dihydropyridines | 4,231 (28.6) | 24,037 (30.3) | 0.032 |
| Low ceiling diuretics | 567 (3.8) | 3,615 (4.6) | 0.030 |
| Beta blockers | 5,023 (33.9) | 30,316 (38.3) | 0.074 |
| Non-hydropyridines | 175 (1.2) | 1,040 (1.3) | 0.010 |
| High ceiling diuretics | 2,024 (13.7) | 15,086 (19.0) | 0.122 |
| Aldosteron antagonists | 695 (4.7) | 4,352 (5.5) | 0.030 |
| Digoxin | 267 (1.8) | 2,771 (3.5) | 0.091 |
| Flekanide | 22 (0.1) | 133 (0.2) | 0.004 |
| Amiodarone | 33 (0.2) | 453 (0.6) | 0.049 |
| Warfarin | 707 (4.8) | 6,160 (7.8) | 0.105 |
| Receptor P2Y12 antagonists | 670 (4.5) | 4,744 (6.0) | 0.055 |
| Direct factor Xa inhibitors | 143 (1.0) | 1,429 (1.8) | 0.062 |
| Direct thrombin inhibitor | 123 (0.8) | 916 (1.2) | 0.028 |
| Other antiplatelets | 137 (0.9) | 1,130 (1.4) | 0.039 |
| Corticosteroids | 683 (4.6) | 5,007 (6.3) | 0.063 |
| Weight loss drugs | 183 (1.2) | 415 (0.5) | 0.058 |

SD, Standard deviation. All numbers in parenthesis are percentage if not stated otherwise.

CV, cardiovascular, CABG, coronary artery bypass grafting; PCI, percutaneous coronary intervention; COPD, chronic obstructive pulmonary disease. DPP-4i, dipeptidyl-peptidase-4 inhibitors; SGLT-2i, Sodium-glucose-cotransporter-2-inhibitors; GLP-1RA, glucagon-like peptide-1 receptor agonists; ACE, angiotensin-converting enzyme; ARB, angiotensin receptor blocker, CVD, Cardiovascular disease

**Supplemental Table 3** — Baseline of propensity matched 1:3 type 2 diabetes patients being new users of dapagliflozin versus dipeptidyl peptidase-4 inhibitor (DPP-4i).

|  | Dapagliflozin | DPP-4i | Standardized difference (%) |
| --- | --- | --- | --- |
| No. of patients | 10227 | 30681 |  |
| Age, years (SD) | 61 (11.1) | 60.8 (12.4) | 0.017 |
| Gender (Female) | 4,196 (41.0) | 12,391 (40.4) | 0.011 |
| First GLD, years | 6.5 (4.1) | 6.5 (4.1) | 0.009 |
| Index year |  |  |  |
| 2012 | 10 (0.1) | 126 (0.4) | 0.057 |
| 2013 | 1,516 (14.8) | 4,565 (14.9) | 0.001 |
| 2014 | 4,152 (40.6) | 12,281 (40.0) | 0.009 |
| 2015 | 4,549 (44.5) | 13,709 (44.7) | 0.003 |
| First GLD year |  |  |  |
| 1995 | 109 (1.1) | 398 (1.3) | 0.018 |
| 1996 | 35 (0.3) | 151 (0.5) | 0.020 |
| 1997 | 42 (0.4) | 128 (0.4) | 0.001 |
| 1998 | 53 (0.5) | 174 (0.6) | 0.005 |
| 1999 | 73 (0.7) | 175 (0.6) | 0.014 |
| 2000 | 78 (0.8) | 216 (0.7) | 0.006 |
| 2001 | 81 (0.8) | 249 (0.8) | 0.002 |
| 2002 | 87 (0.9) | 246 (0.8) | 0.004 |
| 2003 | 97 (0.9) | 319 (1.0) | 0.008 |
| 2004 | 1,259 (12.3) | 3,570 (11.6) | 0.017 |
| 2005 | 1,982 (19.4) | 5,913 (19.3) | 0.002 |
| 2006 | 660 (6.5) | 1,930 (6.3) | 0.005 |
| 2007 | 605 (5.9) | 1,825 (5.9) | 0.001 |
| 2008 | 656 (6.4) | 1,929 (6.3) | 0.004 |
| 2009 | 627 (6.1) | 1,963 (6.4) | 0.009 |
| 2010 | 667 (6.5) | 1,966 (6.4) | 0.004 |
| 2011 | 666 (6.5) | 1,985 (6.5) | 0.001 |
| 2012 | 628 (6.1) | 1,915 (6.2) | 0.003 |
| 2013 | 682 (6.7) | 2,085 (6.8) | 0.004 |
| 2014 | 679 (6.6) | 2,077 (6.8) | 0.004 |
| 2015 | 461 (4.5) | 1,467 (4.8) | 0.011 |
| Frailty | 1,592 (15.6) | 4,799 (15.6) | 0.002 |
| Cardiovascular disease | 2,356 (23.0) | 6,970 (22.7) | 0.006 |
| Myocardial infarction | 730 (7.1) | 2,183 (7.1) | 0.001 |
| Coronary artery bypass grafting | 200 (2.0) | 630 (2.1) | 0.006 |
| Percutaneous Coronay intervention | 583 (5.7) | 1,746 (5.7) | 0.000 |
| Unstable angina | 372 (3.6) | 1,150 (3.7) | 0.005 |
| Angina pectoris | 965 (9.4) | 2,923 (9.5) | 0.003 |
| Heart failure | 498 (4.9) | 1,474 (4.8) | 0.002 |
| Atrial fibrillation | 719 (7.0) | 2,060 (6.7) | 0.010 |
| Stroke | 651 (6.4) | 1,925 (6.3) | 0.003 |
| *Hemorrhagic* | 71 (0.7) | 219 (0.7) | 0.002 |
| *Ischemic* | 421 (4.1) | 1,245 (4.1) | 0.002 |
| *Transitory ischemic attack* | 238 (2.3) | 692 (2.3) | 0.004 |
| Peripheral artery disease | 529 (5.2) | 1,549 (5.0) | 0.005 |
| Major organ specific bleeding | 314 (3.1) | 964 (3.1) | 0.003 |
| Bariatric surgery | 40 (0.4) | 123 (0.4) | 0.001 |
| Chronic kidney disease | 108 (1.1) | 298 (1.0) | 0.007 |
| Dialysis | 9 (0.1) | 27 (0.1) | 0.000 |
| Microvascular complications | 2,172 (21.2) | 6,414 (20.9) | 0.007 |
| *Neuropathy* | 284 (2.8) | 784 (2.6) | 0.011 |
| *Eye complications* | 804 (7.9) | 2,484 (8.1) | 0.007 |
| *Peripheral angiopathy* | 177 (1.7) | 466 (1.5) | 0.014 |
| *Kidney disease* | 187 (1.8) | 600 (2.0) | 0.008 |
| *Several-/unspecified complications* | 1,263 (12.3) | 3,873 (12.6) | 0.007 |
| Severe hypoglycemia | 191 (1.9) | 556 (1.8) | 0.003 |
| Keto-/lactate acidosis | 70 (0.7) | 202 (0.7) | 0.003 |
| Cancer | 648 (6.3) | 1,977 (6.4) | 0.004 |
| COPD | 402 (3.9) | 1,144 (3.7) | 0.009 |
| Lower limb amputations | 37 (0.4) | 102 (0.3) | 0.004 |
| Metformin | 8,522 (83.3) | 25,705 (83.8) | 0.010 |
| Sulphonylurea | 2,668 (26.1) | 7,920 (25.8) | 0.005 |
| GLP-1RA | 798 (7.8) | 2,309 (7.5) | 0.008 |
| Metiglinides | 209 (2.0) | 597 (1.9) | 0.006 |
| Thiazolidinediones | 148 (1.4) | 416 (1.4) | 0.006 |
| Acarbose | 50 (0.5) | 142 (0.5) | 0.003 |
| Insulin | 3,105 (30.4) | 8,920 (29.1) | 0.023 |
| Short-acting | 1,124 (11.0) | 3,307 (10.8) | 0.006 |
| Intermediate-acting | 1,504 (14.7) | 4,358 (14.2) | 0.012 |
| Premixed insulin | 813 (7.9) | 2,350 (7.7) | 0.009 |
| Long-acting | 1,044 (10.2) | 3,062 (10.0) | 0.006 |
| Metformin+Metiglinides | 6 (0.1) | 20 (0.1) | 0.002 |
| CVD risk treatment | 8,702 (85.1) | 26,041 (84.9) | 0.005 |
| Low dose aspirin | 3,497 (34.2) | 10,434 (34.0) | 0.003 |
| Statins | 6,457 (63.1) | 19,405 (63.2) | 0.002 |
| Antihypertensives | 7,483 (73.2) | 22,255 (72.5) | 0.012 |
| ACE inhibitors | 3,108 (30.4) | 9,246 (30.1) | 0.005 |
| ARB | 3,807 (37.2) | 11,261 (36.7) | 0.009 |
| Dihydropyridines | 2,841 (27.8) | 8,467 (27.6) | 0.003 |
| Low ceiling diuretics | 439 (4.3) | 1,292 (4.2) | 0.003 |
| Beta blockers | 3,533 (34.5) | 10,408 (33.9) | 0.011 |
| Non-hydropyridines | 119 (1.2) | 353 (1.2) | 0.001 |
| High ceiling diuretics | 1,364 (13.3) | 4,036 (13.2) | 0.004 |
| Aldosteron antagonists | 441 (4.3) | 1,303 (4.2) | 0.003 |
| Digoxin | 192 (1.9) | 550 (1.8) | 0.005 |
| Flekanide | 18 (0.2) | 50 (0.2) | 0.003 |
| Amiodarone | 30 (0.3) | 78 (0.3) | 0.006 |
| Warfarin | 527 (5.2) | 1,530 (5.0) | 0.006 |
| Receptor P2Y12 antagonists | 471 (4.6) | 1,351 (4.4) | 0.008 |
| Direct factor Xa inhibitors | 104 (1.0) | 304 (1.0) | 0.002 |
| Direct thrombin inhibitor | 78 (0.8) | 228 (0.7) | 0.002 |
| Other antiplatelets | 112 (1.1) | 319 (1.0) | 0.004 |
| Corticosteroids | 478 (4.7) | 1,422 (4.6) | 0.002 |
| Weight loss drugs | 99 (1.0) | 275 (0.9) | 0.006 |

SD, Standard deviation. All numbers in parenthesis are percentage if not stated otherwise.

CV, cardiovascular, CABG, coronary artery bypass grafting; PCI, percutaneous coronary intervention; COPD, chronic obstructive pulmonary disease. DPP-4i, dipeptidyl-peptidase-4 inhibitors; SGLT-2i, Sodium-glucose-cotransporter-2-inhibitors; GLP-1RA, glucagon-like peptide-1 receptor agonists; ACE, angiotensin-converting enzyme; ARB, angiotensin receptor blocker, CVD, Cardiovascular disease

**Supplemental Table 4 —** Weighted means of hazard ratios (HRs) in Denmark, Norway and Sweden for new users of dapagliflozin versus dipeptidyl peptidase-4 inhibitor (DPP-4i) including the follow-up time after index treatment discontinuation (intention to treat) and separate analysis on inpatient hospitalization for heart failure. The groups were matched 1:3 using propensity scores based on age, sex, frailty (three or more days in hospital within one year prior to index) comorbidity and treatment.

|  | **Dapagliflozin**  N=10,227 | | **DPP-4i**  N=30,681 | | **Weighted average estimates**  N=40,908 | | |
| --- | --- | --- | --- | --- | --- | --- | --- |
|  | No. events | Events/100 PYR | No. events | Events/100 PYR | Hazard ratio | 95% CI | p-value |
| **Intention to treat** |  |  |  |  |  |  |  |
| **MACE** | 228 | 2.00 | 813 | 2.35 | 0.85 | (0.74-0.99) | 0.032 |
| Nonfatal myocardial infarction | 111 | 0.97 | 348 | 1.00 | 0.99 | (0.80-1.22) | 0.891 |
| Nonfatal stroke | 89 | 0.78 | 316 | 0.91 | 0.86 | (0.68-1.09) | 0.205 |
| Cardiovascular mortality | 51 | 0.44 | 205 | 0.58 | 0.78 | (0.57-1.06) | 0.114 |
| **HHF** | 123 | 1.08 | 525 | 1.51 | 0.69 | (0.57-0.84) | <0.001 |
| **MACE +** | 256 | 2.25 | 910 | 2.63 | 0.85 | (0.74-0.98) | 0.025 |
| Unstable angina | 44 | 0.38 | 128 | 0.37 | 1.05 | (0.75-1.48) | 0.763 |
| **MACE ++** | 361 | 3.19 | 1337 | 3.91 | 0.81 | (0.72-0.91) | <0.001 |
| **All-cause mortality** | 203 | 1.34 | 905 | 1.97 | 0.69 | (0.60-0.81) | <0.001 |
| **Atrial fibrillation** | 165 | 1.45 | 519 | 1.50 | 0.94 | (0.79-1.12) | 0.518 |
| **Severe hypoglycemia** | 112 | 0.98 | 356 | 1.02 | 0.97 | (0.79-1.20) | 0.785 |
| **On treatment*** |  |  |  |  |  |  |  |
| HHF | 72 | 1.1 | 361 | 1.7 | 0.60 | (0.47-0.78) | <0.001 |
| HHF, inpatient visit only | 32 | 0.5 | 193 | 0.9 | 0.51 | (0.35-0.74) | <0.001 |

HHF, hospital event for heart failure

MACE, major adverse cardiovascular event defined by cardiovascular mortality, myocardial infarction and stroke.

MACE+, addition of unstable angina

MACE++, addition of unstable angina and HHF

*Only Norwegian and Swedish data
